# Mixed-Species Cover Crop Biomass Estimation Using Planet Imagery

**DOI:** 10.3390/s23031541

**Published:** 2023-01-31

**Authors:** Tulsi P. Kharel, Ammar B. Bhandari, Partson Mubvumba, Heather L. Tyler, Reginald S. Fletcher, Krishna N. Reddy

**Affiliations:** Crop Production Systems Research Unit, USDA-ARS, Stoneville, MS 38776, USA

**Keywords:** cover crops, hyperspectral reflectance, remote sensing, machine learning, satellite imagery

## Abstract

Cover crop biomass is helpful for weed and pest control, soil erosion control, nutrient recycling, and overall soil health and crop productivity improvement. These benefits may vary based on cover crop species and their biomass. There is growing interest in the agricultural sector of using remotely sensed imagery to estimate cover crop biomass. Four small plot study sites located at the United States Department of Agriculture Agricultural Research Service, Crop Production Systems Research Unit farm, Stoneville, MS with different cereals, legumes, and their mixture as fall-seeded cover crops were selected for this analysis. A randomized complete block design with four replications was used at all four study sites. Cover crop biomass and canopy-level hyperspectral data were collected at the end of April, just before cover crop termination. High-resolution (3 m) PlanetScope imagery (Dove satellite constellation with PS2.SD and PSB.SD sensors) was collected throughout the cover crop season from November to April in the 2021 and 2022 study cycles. Results showed that mixed cover crop increased biomass production up to 24% higher compared to single species rye. Reflectance bands (blue, green, red and near infrared) and vegetation indices derived from imagery collected during March were more strongly correlated with biomass (*r* = 0–0.74) compared to imagery from November (*r* = 0.01–0.41) and April (*r* = 0.03–0.57), suggesting that the timing of imagery acquisition is important for biomass estimation. The highest correlation was observed with the near-infrared band (*r* = 0.74) during March. The R^2^ for biomass prediction with the random forest model improved from 0.25 to 0.61 when cover crop species/mix information was added along with Planet imagery bands and vegetation indices as biomass predictors. More study with multiple timepoint biomass, hyperspectral, and imagery collection is needed to choose appropriate bands and estimate the biomass of mix cover crop species.

## 1. Introduction

Cover crops (CCs) are promoted as an approach for soil resource conservation while utilizing the land under a sustainable crop production system. Cover crops help to reduce soil erosion during a fallow period [1], recycle nutrients and water [2,3], suppress weed population [4], and add soil organic matter [5,6,7]. Legume cover crops can fix atmospheric nitrogen [8,9], while grass cover crops are better at weed suppression [4,10], organic matter addition and soil erosion control [11]. The mixture of legume and grass balances the C:N ratio on the decomposing materials after cover crop termination, and subsequent crops can utilize nutrients released early in the season. Hence cover crop benefits may vary based on cover crop species, their mixes, and the amount of biomass they produce to cover the ground and to be decomposed [12]. With renewed interest in them, there is potential for increased adoption of cover crops in crop production systems. The United States Department of Agriculture (USDA) Environmental Quality Incentives Program (EQIP) increased cover crop funding support by up to 33% in 2015 compared to 2007 spending, an indication to expect more cover crop adoption in the near future by crop growers. Thus, to quantify cover crop benefits, acreage adoption of different cover crop species/mixes and their biomass production during cover crop season is required.

Remote sensing can be used to efficiently document the adoption and benefits of CC [13], thus reducing the time and the resources needed for extensive field sampling and measurements. Relatively more work has been performed on classifying cover crops at the landscape scale using coarse resolution satellite imagery [14,15,16,17] than on estimating cover crop biomass using fine-scale imagery. Using relatively finer resolution imagery [18] reported that monoculture grass biomass was strongly correlated with SPOT-5 derived normalized difference vegetation index (NDVI) values. Similarly, [19] showed that Sentinel-2 imagery can help assess cover crop growth. However, a within-field biomass estimation requires more precise reflectance characteristics of cover crops. Additionally, work on cover crop biomass estimation using remote sensing approach is more challenging due to the adoption of mix cover crop species by growers, which demands a more detailed reflectance characterization of the mixed species. Hence, estimating biomass yield and their benefit with remote sensing requires reflectance characteristics and identification of appropriate spectral bands and vegetation indices for each CC species and their mix.

Planet Lab (Planet Labs, Inc., San Francisco, CA, USA, https://www.planet.com, accessed on 5 November 2022) provides free daily imagery for the research purpose in 3 m spatial resolution [20] that can be used for plot-level analysis of cover crop studies. The PlanetScope (PS) satellite constellation was launched in phases, with groups of individual small satellites (called smallsats or cubesats) named Dove classic, Dove-R and SuperDove. The three generations of instruments on these satellites are called PS2, PS2.SD and PSB.SD, with blue, green, red and near infrared (NIR) sensor capabilities. The third generation of instrument, PSB.SD, was launched in April 2019, with four additional visible bands (coastal blue, green, yellow and red-edge). Planet imagery undergoes varying levels of processing before it is released for analytical purpose. Ortho Scene-Analytics (Level 3B) surface reflectance imagery is an orthorectified, radiometrically calibrated, and atmospherically corrected product that captures bottom of atmosphere reflectance characteristics [21]. The blue, green, red, and NIR images from these products can be used to derive a variety of vegetation indices (VIs) at 3 m spatial resolution. For research purposes, users can sign up with the National Aeronautics and Space Administration (NASA) Commercial Smallsat Data Acquisition (CSDA) program to access these commercial products free of charge. Through the NASA-CSDA program Planet, data are available to U.S. government federal civil agencies and National Science Foundation funded researchers. Students and researchers from any university can access these data through Planet’s Education and Research Program.

Canopy-level reflectance data collected with the hyperspectral instrument can be helpful in developing reflectance characteristics of single- and mixed-species cover crops. Unlike multispectral sensors, hyperspectral sensors record reflected energy in narrow and contiguous channels. This allows more information to be collected and the reflectance characteristics of the target to be developed. Depending on the instrument type and capacity, data collected range from ultraviolet (UV, ~250 nm) to short-wave infrared (SWIR, ~2500 nm). Usually, wavelengths from visible (VIS) to near infrared (NIR) range (400–700 nm) are used for vegetation estimation based on the absorption of light by leaf pigmentation. Mesophyll cell reflects most of the incident light in NIR (700–1300 nm) range while middle-infrared (MIR, 1300–2500 nm) region of the light spectrum is mostly absorbed by soil and leaf water contents [22]. Hence these absorption and reflectance features of specific portion of the light spectrum are used to develop different vegetation indices. Most vegetation indices developed are closely related to biomass, leaf area index, and photosynthetically active radiation absorption and reported widely for vegetation estimation [22]. Furthermore, because hyperspectral reflectance data are rich in spectral information, they can be used to separate crops into taxonomic units such as species and even in varieties [23,24].

An exploratory analysis of ongoing cover crop research will be presented in this paper. Although biomass estimation at landscape level has been evaluated by several other researchers [13,14,15,16,17,18,19], mixed-species cover crop biomass estimation using plot-level dataset is lacking in the literature. The objective is to evaluate the feasibility of mixed-species cover crop biomass estimation using high-resolution satellite imagery. Specifically, we hypothesize that (1) cover crop biomass production increases with mixed species; (2) Planet imagery collected during later growth stages is more strongly correlated with cover crop biomass; and (3) biomass estimation using remotely sensed data with a machine learning approach is not affected by mixed-species cover crops. The reasoning behind the third hypothesis is that more information will be captured throughout the growing season by different wavebands, accounting for species variability. Planet imagery will be used for this purpose. One-time scanned hyperspectral reflectance data will also be used to compare the model output. Although four different cover crop study sites were combined, the comparison of the hyperspectral data is performed with the aim of developing future research directions, rather than drawing any conclusions per se.

## 2. Materials and Methods

### 2.1. Site and Experiment Description

**Figure 1:**
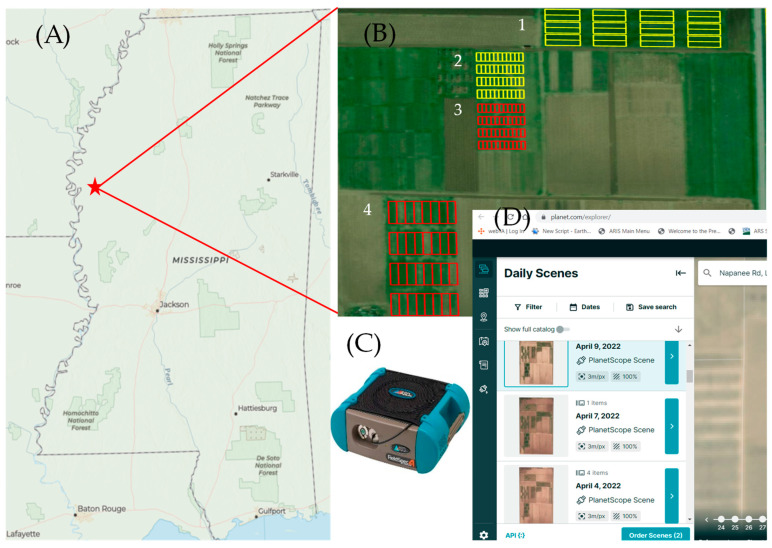
Study site and data products used. (**A**) Mississippi state map with the Crop Production Systems Research Unit (CPSRU) location marked as star; (**B**) four study sites with cover crops (labeled 1–4) within CPSRU research farm (red with main crop corn and yellow with soybean, cover crop treatment detail is provided in Table 1); (**C**) hyperspectral spectroradiometer (Fieldspec4 Standard-Res) used to collect 350–2500 nm canopy reflectance; and (**D**) Planet imagery downloaded for the study site through Planet Explorer.

The study was conducted at the USDA, Agricultural Research Service (ARS), Crop Production Systems Research Unit (CPSRU) research farm located in Stoneville, MS, in the mid-south region of the USA (33°26’36.24’’ N and 90°53’3.08’’ W). Four separate ongoing research study sites (Figure 1) with different cover crop species and seeding rates were selected for this study within the CPSRU research farm. The soil texture within these study sites was predominantly Commerce series (fine-silty, mixed, superactive, nonacid, thermic Fluvaquentic Endoaquepts), with some Dowling series (very-fine, smectitic, nonacid, thermic Vertic Endoaquepts).

Each of the four study sites was established with a randomized complete block design (RCBD) with four replications to evaluate cover crop and other treatments on main crop productivity and soil health. Plots within all four study sites that included cover crop treatments were selected for this paper. Hence, study sites 1, 2, 3, and 4 contained a total of 12, 16, 16, and 32 plots, respectively, with cover crop and fallow treatments (Table 1). Biomass from fallow plots of study sites 2 and 3 were not collected, thus a total of 68 plots (12, 12, 12, and 32 plots for study sites 1, 2, 3, and 4, respectively) were used to perform the analysis for this paper. Study sites 1 and 4 (Figure 1) contained eight rows for each plot (32 m × 8.4 m), while study sites 2 and 3 consisted of four rows per plot (12 m × 4.2 m). All four study sites were planted with cover crop treatments between the last week of October and the first week of November in 2020 and 2021 (Table 1). Study site 1 comprised three cover crop treatments: rye (*Secale cereale* L.), rye + crimson clover (CC, *Trifolium incarnatum* L.), and no winter cover crop, planted both in no-till and conventional till plots. Data from both no-till and conventional till plots were combined in study site 1. The main crop at study site 1 was soybean (*Glycine max* L.), planted a week after cover crop termination. Study sites 2 and 3 contained mixed-species cover crops seeded with different seeding rates (Table 1). Species included were Austrian winter pea (*Pisum sativum* L.), rye, crimson clover, hairy vetch (*Vicia villosa* Roth), wheat (*Triticum aestivum* L.), oats (*Avena sativa* L.), and purple turnip (*Brassica rapa* L.). The main crop at study sites 2 and 3 were soybean and corn (*Zea mays* L.), respectively. Study site 4 included rye, hairy vetch, and crimson clover as individual species and a mix of these cover crop species (Table 1). The main crop at study site 4 was corn. Standard recommended agronomic practices for the main crop (corn and soybean) were followed with fertilizer and herbicide application while cover crop received no fertilizers and herbicides. Cover crops were terminated a week before main crop planting by application of paraquat dichloride.

**Figure 2:**
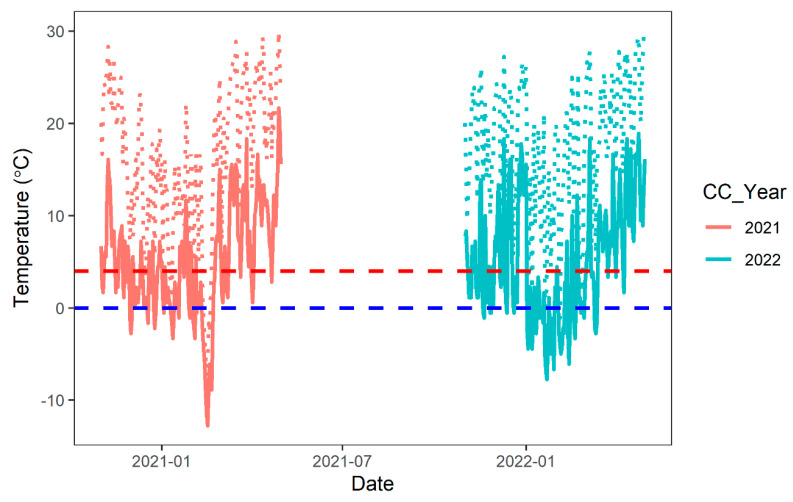
Temperature during 2021 and 2022 cover crop cycles. Solid lines are minimum temperature while dotted lines are maximum temperature. Two dashed lines represent frost period initiation (blue dashed line, 0 °C) and second growing degree day base temperature (red dashed line, 4 °C) for winter cover crops.

Weather data were downloaded from Delta Agricultural Weather Center, Stoneville, MS (2.5 km from study plots) (http://deltaweather.extension.msstate.edu/stoneville-aws, accessed on 20 November 2022). The daily maximum and minimum air temperature from cover crop planting to termination for both 2021 and 2022 were analyzed for cover crop growing degree days (GDD) calculation. Growing degree days were calculated using base 0 and 4 °C (Table 1, Figure 2). All study sites were within an area of 0.5 km^2^, and received the same amount of precipitation throughout the cover crop growing season. The values of monthly total precipitation in the 2021 growing season were 45, 138, 67, 118, 114 and 89 mm, and in the 2022 growing season a39, 46, 43, 117, 156, and 143 mm for November, December, January, February, March and April, respectively.

### 2.2. Biomass and Hyperspectral Data Collection and Processing

Cover crop biomass samples were collected before CC termination in April or May of 2021 and 2022 (Table 1). Biomass was collected within each experimental unit (plot) using two randomly placed 1 m^2^ sampling frames. Above-ground biomass within each frame was clipped, bagged together, dried in the greenhouse for over 2 months, and weighed to determine average dry biomass production per square meter area. Finally, biomass yield from each plot was expressed as Mg ha^−1^ basis.

Hyperspectral reflectance data were collected using FieldSpec4 standard resolution (ASD Inc., Malvern PANalytical, Westborough, MA, USA) spectroradiometer held over the cover crop canopy. This instrument measured wavebands from 350 to 2500 nm from the target area. Cover crop canopies were scanned randomly at 3 different spots within each plot, and averaged reflectance value was used for further analysis. The R [25] package ‘hsdar’ [26] was used to postprocess the hyperspectral data. Hyperspectra were filtered (350–400, 1330–1480, 1780–1900, 2400–2500 nm), smoothed (Savitzky–Golay noise filter), and resampled to 10 nm bands, resulting in 164 total reflectance bands. Vegetation indices for hyperspectral data were derived using *hsdar::vegindex* command (vegindex function from hsdar package). A total of 115 VIs were retrieved and used for the random forest model-building process. A list of VIs, their derivation, and source can be found in R using ‘*help(vegindex)*’ or at the URL: https://rdrr.io/cran/hsdar/man/vegindex.html. Since hyperspectral data were collected at one time point at the end of season, no other statistics except the random forest model output for that specific time point were compared with planet imagery.

### 2.3. Planet Imagery Download and Processing

For both the 2021 and 2022 cover crop cycles, weekly (5–7 days based on cloud coverage on experimental fields) cloud-free Planet imagery was downloaded. The acquisition time period for these Planet data ranged from the approximate planting date (1 November) until CC termination (30 April). Planet imagery was accessed through the NASA-CSDA program. Analysis-ready surface reflectance images (PlanetScope AnalyticMS Level 3B product) were downloaded using Planet Explorer. This imagery had a spatial resolution of 3 m, with blue (464–517 nm), green (547–585 nm), red (650–682 nm) and NIR (846–888 nm) bands. Since all four study sites fell within one of the Planet satellite’s swath size of 24 km × 16 km, no further postprocessing for geometric correction was performed on the downloaded product. A total of 47 Planet images (representing the weekly image of the study sites for two years) were used for further processing and analysis.

Each of the 47 Planet images with their surface reflectance bands (blue, green, red and NIR) were overlaid on the experimental plot boundaries (i.e., the 68 plots at the four study sites), and the pixel values were extracted using the R *raster::extract* function. Imagery pixels that were 50% or more, by area, within the experimental plot boundary were included in the calculation of the average pixel value per plot. Vegetation indices (Table 2) were derived from the plot-level average pixel value of the blue, green, red and NIR bands. Most of the vegetation indices (NDVI), enhanced vegetation index (EVI), two-band enhanced vegetation index (EVI2), optimized soil-adjusted vegetation index (OSAVI), simple ratio (SR), and modified simple ratio (MSR) are closely related to biomass, leaf area index, and photosynthetically active radiation absorption, and have been reported widely for the purpose of vegetation estimation [22,27,28,29,30,31,32,33,34]. Excess green index (ExG) and excess red index (ExR) are, however, used to enhance vegetation features in RGB imagery and count plant density [35,36,37,38] remotely.

Based on the temperature and frost periods observed during the 2021 and 2022 cover crop cycle (Figure 2), the dataset (plot-level bands and VI values) from both years was divided into four time periods for statistical analysis: November, March, April_A (1–15 April), and April_B (15–30 April). For each time period, the average bands and VI values were calculated using the weekly value extracted in the earlier step. Hence, A total of 544 observations of reflectance values of bands and VIs (68 plots × 4 time periods × 2 years) returned by these steps were used for further statistical analysis and model building process.

### 2.4. Statistical Analysis and Random Forest Model Building

All four study sites were combined to explore the correlation (Pearson’s correlation coefficient, r) of seasonal (or time period November, March, April_A, April_B) VI and reflectance bands with cover crop biomass and seeding rate. Based on the strength of correlation observed in our study and importance shown by other literature, red, NIR, NDVI, EVI and OSAVI [22,27,28,31,32] were further analyzed using analysis of variance (ANOVA) to explore the CC treatment effect on band/indices value and biomass yield. Each experiment was analyzed separately for ANOVA, and the least significant difference (LSD) for CC treatment was identified at *p* ≤ 0.05. R software was used to run the ANOVA model, where CC, year, and their interaction were included to extract appropriate mean square error (MSE) to test LSD among CC treatments.

The random forest models were developed using the R package “randomForest’ [39] to predict biomass with planet imagery and hyperspectral data. For Planet imagery, 12 predictors (four reflectance bands and eight VIs) were used to develop a random forest model for each time period (November, March, first half of April and second half of April). In the next step, CC species information was added to the 12 variables to predict biomass. For hyperspectral data, data were only available for one time point (during biomass sampling at the end of April), and models were developed using all bands (164) first, then using all derived VIs (115) to predict biomass. The cover crop species information was added to the bands and VIs to predict biomass in the second step. Data were split into 70% training and 30% testing during each model run. Five-fold cross-validation and grid search criteria were used to fine-tune the model parameter mtry (randomly selected predictor during each split) using the R package “caret” [40]. The model parameter ntree (i.e., the number of trees to grow) was set to 150. The final model was used to test on the remaining 30% dataset and test statistics R^2^ and mean square error (MSE) were calculated.

## 3. Results

### 3.1. Weather and Management Effect on Cover Crop Biomass

**Figure 3:**
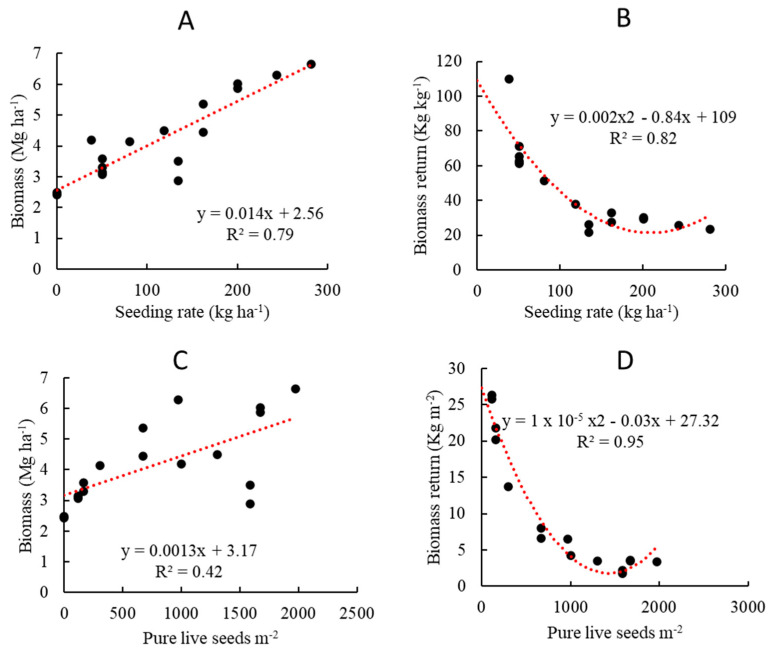
Cover crop seeding rate and pure live seed effect on biomass (across all four studies and cover crop species/mix for both year 2021 and 2022): (**A**) biomass yield and seeding rate linear relationship; (**B**) biomass return per kilogram of seeds; (**C**) biomass and pure live seeds linear relationship; and (**D**) biomass return per square meter of pure live seeds. Least square mean (individual points on the figure) of each treatment was used to plot the relationship and trends.

During the 2021 cover crop cycle, the temperature dropped below freezing (0 °C) first on 30 November 2020, and stayed above freezing after 22 February 2021. During the 2022 CC cycle, the temperature dropped to freezing on 19 November 2021, and it stayed above freezing after 28 February 2022. Growing degree days based on 0 °C base temperature during the 2021 cover crop season were 1908, 1749, 1749 and 1805 for studies 1, 2, 3 and 4, respectively, while they were 2201, 1852, 1852 and 1792 during 2022 (Table 1). The overall relationship between GDD and cover crop biomass across all studies, years, and cover crop species/mixes was very weak (R^2^ < 0.02), regardless of the GDD base temperature, i.e., 0 vs. 4 °C. However, the seeding rate showed a strong linear relationship (R^2^ = 0.79; Figure 3A) with cover crop biomass. Cover crop biomass increased with a higher seeding rate. Biomass return for each unit of seeding rate (Kg biomass per Kg seeding rate, Figure 3B) decreased with higher seeding rate and became stable at around 20–30 kg biomass for each kg of seeds after 135 kg ha^−1^ seeding rate. Biomass also increased with higher number of pure live seeds (PLS) per square meter (Figure 3C). Biomass return per square meter of PLS decreased with a higher number of PLS per square meter and became stable at 3.5 kg m^−2^ once PLS reached approximately 1300 (Figure 3D).

### 3.2. Planet Imagery and Cover Crop Biomass Relationship

Imagery bands and indices from March and April were better correlated with cover crop biomass compared to November (Table 3). Generally, the correlation was highest in March, and the NIR data were most strongly correlated (*r* = 0.74) with biomass during this time period. Within the bands (blue, green, red, and NIR), NIR was the only one strongly and positively correlated with biomass during March. However, out of the eight VIs (Table 2) explored in this study, five VIs (NDVI, EVI, EVI2, OSAVI, and SR) were positively correlated with CC biomass (Table 3). These five VIs again showed a significant correlation with biomass during the first half of April; however, their coefficient decreased by this time period, except for NDVI. Correlation further decreased for these five VIs by the second half of April, and these VIs were no longer statistically correlated with biomass. Biomass correlation with the visible bands (blue, green, and red) increased in April compared to November and March. Biomass correlation with MSR increased during the latter part of the cover crop season, and it was the only VI correlated during the second half of April.

Since biomass and seeding rate were highly correlated (Figure 3), we tested another hypothesis that the seeding rate was related to bands and VIs. Correlation with seeding rate (Table 3) showed that the same five VIs (NDVI, EVI, EVI2, OSAVI, and SR) and NIR were correlated with biomass and seeding rate during March.

### 3.3. Planet Imagery and Cover Crop Biomass—ANOVA

The four study sites had different cover crop species and mixed combination treatments. Their biomass production ranged from 2.48 Mg ha^−1^ for study site 1, fallow plot, to 6.65 Mg ha^−1^ for study site 4, rye + clover + vetch cover crop combination (Table 4). Only two study sites (1 and 4) included biomass measurements from fallow plots, where weeds with random mixture of swinecress (*Coronopus didymus* (L.) *Sm.*), hairy buttercup (*Ranunculus sardous* Crantz), henbit (*Lamium amplexicaule* L.), white clover (*Trifolium repens* L.), and Italian ryegrass (*Lolium perenne* L. ssp. *multiflorum (Lam.) Husnot*) were observed. Weeds were completely suppressed in the cover crop plots. Overall, higher biomass was observed at study sites 1 (5.17 Mg ha^−1^) and 4 (5.31 Mg ha^−1^) compared to study sites 2 (3.20 Mg ha^−1^) and 3 (3.30 Mg ha^−1^), where fallow plots were excluded.

At study site 1, the rye cover crop produced 80% more biomass than the fallow plot. Rye + clover mix produced 32% more biomass compared to the rye cover crop. Even though biomass differed by CC treatment, none of the reflectance bands and indices were affected by these biomass differences. At study site 2, neither cover crop biomass nor any reflectance bands and indices were affected by cover crop treatment. At study site 3, the low seeding rate (50 kg ha^−1^) of legume mix and cereal mix produced 3.1 and 3.3 Mg ha^−1^ biomass, respectively; however, they were not statistically significantly different. The high seeding rate (135 kg ha^−1^) of legume + cereal mix (HiMix-LC treatment) produced 3.5 Mg ha^−1^ biomass, and it was statistically significantly different from low seeding rate legume mix only. The canopy reflectance index EVI showed the highest value with legume mixed and the lowest with legume/cereal mix. No other VIs and bands were affected by CC species/mix.

Hairy vetch produced the lowest biomass (4.14 Mg ha^−1^) from cover crop treatments (other than fallow plot) at study site 4. Biomass was similar for clover, vetch, and clover + vetch cover crop treatments. However, rye and its mix with legumes (clover and vetch) produced higher biomass. Compared to rye cover crops, rye + clover and rye + vetch produced 12.3% and 17.3% more biomass, respectively. Compared to rye, rye + clover + vetch produced 24% more biomass. On average, legume-based cover crops (clover, vetch, and clover + vetch) produced 4.28 Mg ha^−1^ biomass, while legume mixed with rye (rye + clover, rye + vetch and rye + clover + vetch) produced 6.32 Mg ha^−1^ biomass. Canopy reflectance and VIs were highly correlated with biomass at study site 4, with the red, NIR, NDVI, EVI and OSAVI Pearson correlation coefficients (r) being −0.73, 0.79, 0.68, 0.70 and 0.70, respectively. Reflectance in red bands was highest in the fallow plot, followed by rye cover crops, and lowest in the plots with rye + clover + vetch where biomass was highest. Reflectance in the NIR band was opposite to red and was highest in rye +clover + vetch plots and lowest in fallow plots. Vegetation indices NDVI, EVI, and OSAVI showed a similar pattern to that of NIR, with the highest value being in rye + clover + vetch plots and lowest values in fallow followed by rye cover crop plots.

### 3.4. Biomass Prediction Using Random Forest Model

Biomass prediction using Planet imagery was evaluated for each time period (November, March, first half of April, and second half of April). The coefficient of determination (R^2^) was low when bands and VIs were used as predictors (Table 5). Across the time period evaluated in this study, R^2^ was highest during March and lowest during the second half of April. The model predicted better when CC species/mix information was also added as a predictor. During March, including CC as the predictor increased R^2^ from 0.25 to 0.61, and it decreased MSE from 2.72 to 1.51.

The timing of hyperspectral data collection corresponded to the April_B time period for the Planet imagery data. Biomass predictability from Planet imagery during the April_B time period was very weak, and R2 improved from 0 to 0.36 when using CC species information in the model. FieldSpec showed better biomass predictability (R^2^ 0.35 for bands and 0.46 for VIs). Therefore, the comparison between Planet imagery model output during the April_B time period and the hyperspectral model showed that biomass prediction was better with hyperspectral data compared to Planet imagery (Table 5), probably due to the higher number of bands included in the model and the greater amount of information in these bands. In addition, biomass prediction was better with VIs than bands (R^2^ 0.35 vs. 0.46). Unlike Planet imagery, the addition of CC information had no impact on biomass prediction.

**Figure 4:**
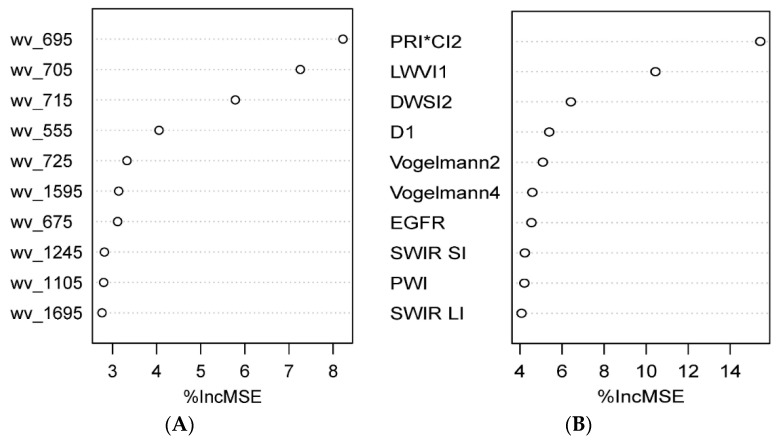
Random forest model selection of the most important (**A**) ten out of 164 wavebands and (**B**) ten out of 115 vegetation indices for predicting CC biomass using hyperspectral data. (wv = wavebands, %incMSE = % increase in mean square error (MSE)).

The ten most important wave bands for predicting cover crop biomass ranged from 555 nm to 1695 nm (Figure 4A). Among the tested VIs (Figure 4B), the ten most important variables were photochemical reflectance index (PRI) × carotenoid index (CI) [41], leaf water vegetation index (LWVI1) [42], disease–water stress index (DWSI-2) [43], derivative index (D1) [44], Vogelmann indices 2 and 4 [45], edge green first derivative ratio (EGFR) [46], shortwave infrared (SWIR SI) [47], plant water index (PWI) [48], and shortwave infrared (SWIR LI) [47]. Since the hyperspectral data were collected once, the authors would like to list only the important variables picked by the random forest model at this stage, rather than to draw further conclusions based on this dataset.

## 4. Discussion

The relationship between seeding rate and biomass yield was explored to test the first hypothesis. A strong linear relationship exists between seeding rate and cover crop biomass regardless of whether single- or mixed-species cover crop planting is employed (Figure 3A). Biomass increase up to a certain seeding rate was expected, but we observed a linear increase in biomass up until the highest seeding rate, 280 kg ha^−1^, in our study. A combination of seeding mixes was used at four study sites where multi-species mixtures were kept at low and high seeding rates (Table 1, Study sites 2 and 3). While seeding rates were not adjusted from single species to mixed species, and 100% rate of single species were added during the mix (study sites 1 and 4), we observed biomass increase with the mixed species. When reducing seeding rate with the mixed-species cover crop treatment [49], it was observed that the biomass produced with mixed species was similar to or on par with the highest biomass produced with single species. The rate of biomass returns per kg of seeds decreased with a higher seeding rate, and a stable return of around 20–30 kg biomass per kg of seeds was observed after a seeding rate of 135 kg ha^−1^ (Figure 3B). Pure live seed counts per square meter of area differ with different cover crop species and could provide a more realistic evaluation compared to seed weight. Biomass was also positively correlated with PLS m^−2^ (Figure 3C). The rate of return of biomass by PLS m^−2^ decreased with a higher number of PLS and became stable around 1300 PLS m^−2^. The PLS count of single cover crop rye was 670 m^−2^ (163 kg ha^−1^ seeds and 85% germination percentage), while rye + clover + vetch was 1972 m^−2^. This result supports mixed cover crop being a better option for higher biomass production and supports our first hypothesis that cover crop biomass increases with mixed species. It was reported in [50] that mixed species produced 1.7 times more biomass than single-species monoculture due to the complementarity effect. therefore, seeding rate recommendations on mixed species should consider not only the biomass produced by a higher seeding rate, but also an economic analysis associated with seed cost, as well as ecological service and functional performance as described by [49] for effective nutrient, moisture, weed suppression and carbon addition to the cropping system. Due to the smaller dataset (fewer study sites, similar planting date, and harvest date), the relationship between GDD and biomass reported in this study is not conclusive.

Our second hypothesis was evaluated using the correlation strength of bands and VIs collected during different time periods with biomass yield. Across the study sites, cover crop biomass correlation with bands and VIs during the month of March was higher compared to that in November and April. During the month of November, ground cover by the cover crop was not sufficient due to the emergence and the small cover crop size, while during April, the reproductive stage (flowering, heading, and leaf yellowing due to translocation of photosynthates from leaves to seeds) with mixed-species cover crops might have contributed to the lower correlation. Vegetation index values increased with time from November to the first half of April, and decreased in the second half of April; however, the highest correlation was observed with VIs derived from the March imagery. Therefore, our second hypothesis—Planet imagery collected during the later growth stage is more strongly correlated with cover crop biomass—was rejected, although the trend showed a relationship until the month of March. Periodic cover crop biomass sampling and its relationship with the VIs will be explored in future work to make a more conclusive argument about the timing of imagery that best correlates with bands and VIs.

Our third hypothesis investigated by testing whether mixed cover crop species affect the remote sensing-based biomass estimation using machine learning approach. By applying the random forest machine learning approach, cover crop biomass was estimated. Cover crop biomass estimation using Planet imagery bands and VIs was not satisfactory, as the highest R^2^ value observed was 0.25 using March imagery (Table 5). This was probably due to the mixed species used in our study. Several authors have reported higher cover crop biomass predictability when using remote sensing indices in single-species studies [18,51,52]. Higher R^2^ values ranging from 0.53 to 0.76 for mixed cover crop species was reported by [53] using Planet imagery. They used several large fields to predict cover crop biomass compared to small plots in the current study, where few pixels were inside each plot. One obvious pattern we observed was the improvement of biomass predictability with the random forest model when CC species information was provided to the model (Table 5). This implies that biomass prediction can be improved if cover crop species are classified beforehand and used as model predictors. Different classification algorithms are developing over time with the availability of higher-resolution imagery and computing power [54,55]. These algorithms can be utilized in automated classification of cover crop species before using a machine learning approach for biomass estimation. Additionally, model predictability improves if more ancillary information is provided. Information such as estimated sowing date from Sentinel imagery helped to explain 55% of the variability in winter cover crop NDVI values [19]. Based on the Planet dataset, our third hypothesis was rejected, and biomass estimation was affected by mixed cover crop species. A previous study using Landsat-8, Sentinel-2 and PlanetScope sensors reported that VIs from PlanetScope sensors were most accurate (lower root mean square error and higher R^2^) for mixed cover crop biomass estimation [53] when individual sampling sites within the fields were considered.

We extended our work with the hyperspectral dataset to test the third hypothesis further. Biomass estimation using hyperspectral bands and indices was better compared to Planet imagery during April (Table 5), but prediction did not improve with the addition of CC species information to the set of predictors. This is likely due to the range of wavebands, and VIs used on the model being enough to capture the total variability of the dataset. This result shows that our third hypothesis was accepted based on the hyperspectral data. However, more hyperspectral studies with measurements throughout the growing season are needed to confirm this result. The hyperspectral model could improve if reflectance data throughout the growing season are captured and utilized in the model-building process, which is a future work target aimed for in this project. The most important bands and VIs from the hyperspectral models were further explored (Figure 4). The bands selected by random forest models from hyperspectral data showed important bands ranging from 555 nm to 1695 nm. Vegetation indices selected by the hyperspectral model utilized bands ranging from 500 nm to 2210 nm. For example, vegetation index EGFR utilizes the derivative of different wavebands from 500 to 750 nm, while SWIR LI and SWIR SI utilize a differential ratio of wavebands 2210 nm and 2090 nm. Wavebands and VIs may be different if the scanning is conducted during the month of March, which is the most highly correlated time period based on Planet imagery.

## 5. Conclusions

An exploratory analysis was conducted to estimate mixed-species cover crop biomass using higher-resolution satellite imagery from the PlanetScope lab and canopy-level hyperspectral reflectance data captured using the FieldSpec4 instrument. Four field study sites with different cover crop species and their mix were analyzed in this paper. Cover crop biomass increased linearly with a higher seeding rate (R^2^ = 0.79) and pure live seeds (R^2^ = 0.42), where a higher seeding rate primarily indicated a mixture of cover crops from the grass and legume family. The results showed that mixed cover crops increased biomass production by 24% compared to single species rye. Evaluation of biomass yield and Planet imagery-based VIs showed that VIs increased over time, but their correlation with biomass yield decreased after March. This result indicates that March (*r* = 0–0.74) is the best time to predict cover crop biomass compared to November (*r* = 0.01–0.41) and April (*r* = 0.03–0.57). Cover crop biomass estimation using the random forest model using the VIs and bands derived from Planet imagery collected during the month of March was below expectation, resulting in an R^2^ value of 0.25. However, the biomass predictability improved (R^2^ = 0.61) with the addition of cover crop species to the model predictors. This result suggests that cover crop biomass estimation is affected by mixed species, and estimation can be improved if species and their mix are classified and used in the model-building process. Contrary to this result, further examination of the same hypothesis with hyperspectral data showed that biomass estimation is not affected by mixed-species due to more biomass variability captured by the increased number of bands used in the model. However, more study with multiple-timepoint biomass, hyperspectral, and imagery collection is needed to choose appropriate bands and predict the biomass of mixed cover crop species.

## Figures and Tables

**Table 1:** Description of study sites and sampling dates.

Study Site	Treatment	Seeding Rate	Planting	Biomass Sampling	FieldSpec Scanning	GDD (0 °C)	GDD (4 °C)
		Kg ha^−1^	Date	Date	Date	Days	Days
1	R *	162	*4 November 2021*	*28 April 2021*		*1908*	1269 **
	R + C	200	27 October 2022	4 May 2022	28 April 2022	2201	1515
	F	0					
2, 3	LoMix-C	50	*6 November 2021*	*21 April 2021*		*1749*	*1147*
	LoMix-L	50	8 November 2022	22 April 2022	27 April 2022	1852	1250
	HiMix-LC	135					
	F	0					
4	R	162	*3 November 2021*	*22 April 2021*		1805	1186
	C	38	9 November 2022	20 April 2022	28 April 2022	1792	1202
	V	80					
	R + C	200					
	R + V	242					
	C + V	118					
	R + C + V	280					
	F	0					

* Cover crop species: R = rye; C = crimson clover; F = fallow; V = hairy vetch; LowMix-C = wheat + rye + black oats; LowMix-L = Austrian winter pea + crimson clover + hairy vetch; HiMix-LC = wheat + rye + black oats + Austrian winter pea + crimson clover + hairy vetch + berseem clover + turnip. ** Planting date, biomass sampling, growing degree days (GDD) and Fieldspec scanning dates in italics are for 2021 and non-italics are for 2022 cover crop cycle. GDD is reported for both base temperature 0 °C and 4 °C.

**Table 2:** Vegetation indices (VIs) used for cover crop biomass estimation. The indices were derived using the blue (B), green (G), red (R), and near-infrared (NIR) bands of Planet imagery.

VIs	Expression #	Source
Normalized difference vegetation index (NDVI)	$\frac{\mathrm{NIR}-R}{\mathrm{NIR}+R}$	[25]
Enhanced vegetation index (EVI)	$2.5*\left(\frac{\mathrm{NIR}-R}{\mathrm{NIR}+6*R-7.5*B+1}\right)$	[26]
Two-band enhanced vegetation index (EVI2)	$2.4*\left(\frac{\mathrm{NIR}-R}{\mathrm{NIR}+2.4*R+1}\right)$	[27,28]
Optimized soil adjusted vegetation index (OSAVI)	$\left(1+0.16\right)*\frac{\left(\mathrm{NIR}-R\right)}{\mathrm{NIR}+R+0.16}$	[29,30]
Simple ratio (SR)	$\frac{\mathrm{NIR}}{R}$	[31]
Modified simple ratio (MSR)	$\frac{\frac{\mathrm{NIR}}{R-1}}{\sqrt{\frac{\mathrm{NIR}}{R}+1}}$	[32]
Excess green index (ExG)	2∗G−R−B	[33,34]
Excess red index (ExR)	1.4∗R−G	[33,35]

# Note: Coefficients of EVI: gain factor G is 2.5, soil and canopy background adjustment factor L is 1, aerosol scattering factor C1 is 6 and C2 is 7.5. Coefficients of EVI2: gain factor G is 2.4, soil and canopy background adjustment factor L is 1, aerosol scattering factor C1 is 2.4. Coefficients of OSAVI: canopy background adjustment factor L is 0.16.

**Table 3:** Pearson correlation coefficients of PlanetScope bands and vegetation indices (VI) during November (Nov), March, and April (April_A = 1–15 April; April_B = 15–30 April) with cover crop biomass and seeding rate.

Bands and VIs	Nov	March	April_A	April_B	Nov	March	April_A	April_B
	Correlation with biomass				Correlation with seeding rate			
	-------------------- r ---------------------------				---------------------- r -----------------------			
Blue	−0.01	−0.34	−0.54 *	−0.54 *	−0.03	−0.35	−0.48	−0.47
Green	−0.15	−0.24	−0.54 *	−0.54 *	−0.11	−0.26	−0.48	−0.48
Red	0.02	−0.42	−0.53 *	−0.42	−0.02	−0.42	−0.46	−0.40
NIR	0.41	0.74 *	0.55 *	0.39	0.26	0.57 *	0.39	0.24
NDVI	0.21	0.56 *	0.57 *	0.43	0.13	0.52 *	0.46	0.34
EVI	0.32	0.66 *	0.57 *	0.44	0.21	0.57 *	0.42	0.35
EVI2	0.41	0.62 *	0.57 *	0.43	0.25	0.52 *	0.43	0.33
MSR	−0.13	0.00	0.27	0.50 *	−0.05	0.24	0.33	0.43
OSAVI	0.35	0.61 *	0.57 *	0.42	0.23	0.54 *	0.44	0.31
SR	0.20	0.60 *	0.56 *	0.45	0.13	0.56 *	0.45	0.34
ExG	−0.35	0.33	0.25	0.03	−0.23	0.21	0.16	0.03
ExR	0.08	−0.44	−0.47	−0.31	0.06	−0.48	−0.45	−0.31

Note: refer to Table 2 for VI derivation information. * Statistically significant at *p* ≤ 0.05.

**Table 4:** Least square means of selected bands and indices from Planet imagery during the month of March and cover crop biomass after analysis of variance (ANOVA).

CC	Seeding	Biomass	Red	NIR	NDVI	EVI	OSAVI
	Kg ha^−1^	Mg ha^−1^	%	%			
**Study site 1**							
R	163	4.45 ^b^	0.07 ^a^	0.33 ^a^	0.64 ^a^	0.45 ^a^	0.46 ^a^
R + C	201	5.88 ^a^	0.07 ^a^	0.33 ^a^	0.63 ^a^	0.44 ^a^	0.45 ^a^
F	0	2.48 ^c^	0.07 ^a^	0.32 ^a^	0.63 ^a^	0.44 ^a^	0.45 ^a^
**Study site 2**							
LoMix-L	50	3.15 ^a^	0.08 ^a^	0.31 ^a^	0.59 ^a^	0.40 ^a^	0.42 ^a^
LoMix-C	50	3.58 ^a^	0.08 ^a^	0.30 ^a^	0.57 ^a^	0.39 ^a^	0.40 ^a^
HiMix-LC	135	2.88 ^a^	0.08 ^a^	0.30 ^a^	0.58 ^a^	0.40 ^a^	0.41 ^a^
**Study site 3**							
LoMix-L	50	3.08 ^b^	0.09 ^a^	0.30 ^a^	0.54 ^a^	0.37 ^a^	0.39 ^a^
LoMix-C	50	3.30 ^ab^	0.09 ^a^	0.30 ^a^	0.52 ^a^	0.36 ^ab^	0.37 ^a^
HiMix-LC	135	3.50 ^a^	0.09 ^a^	0.29 ^b^	0.52 ^a^	0.35 ^b^	0.37 ^a^
**Study site 4**							
R	163	5.36 ^bc^	0.09 ^a^	0.33 ^ab^	0.57 ^cd^	0.40 ^bc^	0.41 ^cd^
C	38	4.19 ^d^	0.08 ^b^	0.33 ^ab^	0.59 ^bc^	0.43 ^ab^	0.43 ^bc^
V	81	4.14 ^d^	0.08 ^b^	0.34 ^a^	0.62 ^ab^	0.45 ^ab^	0.45 ^ab^
R + C	201	6.02 ^ab^	0.08 ^b^	0.34 ^a^	0.61 ^abc^	0.45 ^ab^	0.45 ^ab^
R + V	243	6.29 ^a^	0.08 ^b^	0.34 ^a^	0.61 ^abc^	0.45 ^ab^	0.44 ^abc^
C + V	119	4.5 ^cd^	0.08 ^b^	0.34 ^a^	0.63 ^ab^	0.46 ^a^	0.45 ^ab^
R + C + V	281	6.65 ^a^	0.07 ^c^	0.35 ^a^	0.65 _a_	0.48 ^a^	0.47 ^a^
F	0	2.42 ^e^	0.10 ^a^	0.32 ^b^	0.53 ^d^	0.37 ^c^	0.38 ^d^

Note: Cover crop species/mix with different subscript letters within columns for each study are significant at *p* ≤ 0.05.

**Table 5:** Cover crop biomass prediction using random forest model.

Data Source	Time Period	Predictors	# of Predictors	R^2^	MSE
Planet Imagery	November	Bands + VI	12	0.12	2.60
Planet Imagery	March	Bands + VI	12	0.25	2.72
Planet Imagery	April_A	Bands + VI	12	0.12	3.03
Planet Imagery	April_B	Bands + VI	12	0.00	2.98
Planet Imagery	November	Bands + VI + CC	13	0.60	1.26
Planet Imagery	March	Bands + VI + CC	13	0.61	1.50
Planet Imagery	April_A	Bands + VI + CC	13	0.57	1.51
Planet Imagery	April_B	Bands + VI + CC	13	0.36	1.50
FieldSpec	April	Bands	164	0.35	2.9
FieldSpec	April	Bands + CC	165	0.36	1.23
FieldSpec	April	VI	115	0.46	1.26
FieldSpec	April	VI + CC	116	0.44	0.79

Note: For Planet imagery, November, March, April_A (1–15 April) and April_B (1–15 April) represent average value for that time period, while FieldSpec data were a one-time measurement.

## Data Availability

Data presented are available on request.
